# Supplementary material for: Clinical Characteristics and Prognosis of Older Patients with Coronavirus Disease 2019 Requiring Mechanical Ventilation
Source: J Pers Med. 2024 Jun 19;14(6):657. doi: 10.3390/jpm14060657 (PMC11204506; doi:10.3390/jpm14060657)
Supplement: Supplementary file 1 [file jpm-14-00657-s001.zip › jpm-3037392-supplementary.pdf]

Supplementary Materials

Table S1. Participating hospital name.

	Hospital	Name
1	Pusan National University Yangsan Hospital	Woo Hyun Cho
2	Asan Medical Center	Huh Jin-Won
3	Chung-ang University hospital	Moon Seong Baek
4	Severance hospital	Su Hwan Lee
5	Seoul national university hospital	Sang-Min Lee
6	Samsung Medical Center	Chi Ryang Chung
7	The Catholic University, ST. Mary's Hospital	Jongmin Lee
8	Inha University Hospital	Jung Soo Kim
9	Seoul National University Bundang Hospital	Sung Yoon Lim
10	Soonchunhyang University Bucheon Hospital	Ae-Rin Baek
11	Hallym University Medical Center	Sunghoon Park
12	Gyeongsang National University Hospital	Jung-Wan Yoo
13	Gyeongsang National University Changwon Hospital	Ho Cheol Kim
14	Yeungnam University Hospital	Eun Young Choi
15	Wonkang University Hospital	Chul Park
16	Chonnam National University Hospital	TaeOk Kim
17	Chosun University Hospital	Do Sik Moon
18	Chungnam National University Hospital	Song I Lee
19	Chungnam National University Sejong Hospital	Jae Young Moon
20	Konyang University Hospital	Sun Jung Kwon
21	Jeju National University Hospital	Gil Myeong Seong
22	Korea University Anam Hospital	Won Jai Jung

Table S2. Initial vital sign and laboratory findings of enrolled patients.

Variables	Total Patients (*n* = 434)	Older Patients (*n* = 294)	Very Older Patients (*n* = 140)	*p*-Value
Initial vital sign				
Systolic BP, mmHg	134.8 ± 24.8	133.6 ± 22.6	137.5 ± 28.8	0.161
Diastolic BP, mmHg	74.3 ± 14.8	75.7 ± 14.7	71.4 ± 14.7	0.004
Heart rate, /min	77.6 ± 25.5	77.3 ± 26.9	78.1 ± 22.5	0.768
Respiratory rate, /min	23.4 ± 5.9	23.7 ± 6.3	22.8 ± 5.0	0.107
Body temperature (℃)	36.9 ± 0.8	37.0 ± 0.8	36.9 ± 0.8	0.274
GCS	13.2 ± 3.6	13.4 ± 3.5	12.7 ± 3.7	0.045
Laboratory findings				
White blood cell, 10^3/uL	9.60 ± 8.31	9.37 ± 9.05	10.08 ± 6.49	0.401
Hemoglobin, g/dL	12.6 ± 1.9	12.8 ± 2.0	12.2 ± 1.8	0.004
Platelet, 10^3/uL	184.3 ± 78.0	184.7 ± 74.7	183.5 ± 84.7	0.872
Albumin, g/dL	3.2 ± 0.5	3.2 ± 0.5	3.1 ± 0.5	0.579
Bilirubin, mg/dL	0.72 ± 0.52	0.71 ± 0.53	0.73 ± 0.49	0.737
BUN, mg/dL	26.7 ± 17.9	24.8 ± 15.7	30.7 ± 21.4	0.004
Creatinine, mg/dL	1.20 ± 1.23	1.11 ± 1.13	1.39 ± 1.39	0.040
PT, sec	13.0 ± 3.6	12.9 ± 3.0	13.3 ± 4.5	0.315
C-reactive protein, mg/dL	10.5 (4.9 – 17.6)	10.1 (4.8 – 17.8)	10.6 (5.0 – 16.9)	0.324
Arterial blood gas analysis				
pH	7.42 ± 0.09	7.42 ± 0.8	7.40 ± 0.10	0.019
P/F ratio, mmHg	147.9 ± 95.2	145.5 ± 97.1	153.0 ± 91.0	0.453
Lactate, mmol/L	1.7 (1.2 – 2.3)	1.7 (1.2 – 2.3)	1.9 (1.5 – 2.6)	0.043

Data are presented as mean ± standard deviation or median and interquartile range, unless otherwise indicated. P/F ratio: arterial partial pressure of oxygen/inspired oxygen concentration ratio.
